# Nano-biomaterials in cancer therapy: Advances in targeting and overcoming cancer stem cell resistance

**DOI:** 10.5599/admet.3007

**Published:** 2025-10-24

**Authors:** Marzieh Dehghani, Behnam Hajipour-Verdom, Fatemeh Rahimzadeh, Parviz Abdolmaleki

**Affiliations:** 1 Department of Biophysics, Faculty of Biological Sciences, Tarbiat Modares University, Tehran, 14115-154, Iran; 2 Integrative Oncology Department, Breast Cancer Research Center, Motamed Cancer Institute, Academic Center for Education, Culture and Research (ACECR), Tehran, 1517964311, Iran; 3 Faculty of Medicine and Health, University of Sydney, Sydney, NSW 2015, Australia

**Keywords:** Nanocarriers, therapeutic resistance, drug delivery systems, nanomedicine applications, tumour microenvironment

## Abstract

**Background and purpose:**

Cancer remains a leading cause of death globally, with long-term treatment success hindered by metastasis, recurrence, and therapy resistance. A central driver of these limitations is cancer stem cells (CSCs) - a rare subpopulation of tumour cells endowed with self-renewal and differentiation potential. CSCs contribute critically to tumour progression, metastatic spread, and resistance to conventional therapies, yet no clinically validated strategies currently exist to accurately detect or selectively eliminate them.

**Review approach:**

The paper systematically examined the literature on CSC origin, phenotypic characterization, isolation techniques, and mechanisms underlying resistance, including dormancy, enhanced DNA repair, apoptosis evasion, and microenvironmental protection. Particular attention was given to recent advances in nanomaterial-based strategies - metallic, carbon, and organic nanocarriers, designed to improve CSC-specific drug delivery, reactive oxygen species generation, pathway inhibition, and modulation of the CSC niche.

**Key results:**

Current CSC markers and in vivo models remain ambiguous and poorly standardized, limiting translational progress. Nanotechnology provides promising solutions by enabling targeted delivery and multi-functional therapy integration, yet most systems are still at the preclinical stage, constrained by issues of biocompatibility, targeting precision, and manufacturing scalability.

**Conclusion:**

Multifunctional nanoplatforms hold substantial potential to overcome CSC-driven resistance, improve therapeutic selectivity, and reduce recurrence. However, rigorous optimization and clinical validation are essential before these technologies can be integrated into routine oncology. This review advances understanding by outlining the intersection of CSC biology and nanomedicine, emphasizing translational pathways for CSC-targeted cancer therapy.

## Introduction

Cancer is a major threat to human health, responsible for about 1 in every 6 deaths globally [1]. In 2024, it is estimated that there will be 2 001 140 new cancer cases and 611 720 cancer-related deaths in the United States alone [2]. Despite advances in conventional cancer therapies such as surgery, radiation, and chemotherapy, these treatments often fail to eradicate tumours, especially in aggressive and metastatic forms [3]. The primary reasons for treatment failure include metastasis, recurrence, tumour heterogeneity, and resistance to chemotherapy and radiation. All of these are linked to the presence of cancer stem cells (CSCs), also known as cancer-initiating or tumour-initiating cells, within the tumour microenvironment (TME) [4].

CSCs were first identified in the late 1990s by John Dick in cases of acute myeloid leukaemia [5]. Like normal stem cells, CSCs exhibit self-renewal and differentiation capabilities, enabling them to generate diverse tumour cell populations. These properties render CSCs highly tumorigenic and inherently resistant to conventional therapies [6]. They persist as a distinct population within tumours, contributing to recurrence and metastasis by forming new tumours [7]. Also, a major challenge in targeting CSCs is the lack of universally specific and exclusive biomarkers that can reliably distinguish CSCs from normal stem cells and other tumour cells. While surface markers such as CD44, CD133, aldehyde dehydrogenase 1 (ALDH1), and epithelial cell adhesion molecule (EpCAM) have been widely used to identify CSCs across various tumour types, they are not unique to CSCs and are often expressed in normal progenitor or differentiated cells as well [8]. This overlap hampers the development of precise diagnostic tools and therapeutic strategies [9]. As a result, there is a pressing need to discover and validate novel CSC-specific markers or marker combinations that can improve the specificity and sensitivity of CSC detection.

Therefore, developing treatments that specifically target CSCs offers promising potential to improve both survival and quality of life for cancer patients, especially those with metastatic disease. The field of nanotechnology has opened new avenues in cancer treatment, particularly in cancer nanomedicine. Over the past decade, various organic and inorganic nano-biomaterials have been developed for the diagnosis and treatment of cancer [10]. Their nanoscale size, high surface-to-volume ratio, and tuneable physicochemical properties make nano-biomaterials uniquely suited for biomedical applications. These platforms have enabled more precise drug delivery, enhanced imaging, and selective targeting of CSCs [11].

Natural-based biomaterials play a significant role in cellular and molecular biology, tissue engineering, regenerative medicine, and material science. Micelles, dendrimers, hydrogels, liposomes, and self-assembled polymeric materials are commonly used as nanocarriers to deliver anti-cancer drugs. Both synthetic polymers, such as polyethylene glycol (PEG) and polyvinyl alcohol, and natural polymers, like gelatine, silk fibroin, and collagen, are employed in the synthesis of nano-biomaterials [12]. Additionally, bioactive compounds such as curcumin, gallic acid, and epicatechin have been integrated into these systems. Due to their excellent biocompatibility and multifunctionality, such biomaterials are promising candidates for CSC-targeted therapies [13]. This review aims to explore the biology and therapeutic resistance mechanisms of CSCs and to highlight the current limitations in their detection and elimination. It also evaluates emerging nano-biomaterial-based strategies explicitly designed for CSC targeting. The goal is to provide comprehensive insights into how nanotechnology-based platforms may overcome CSC-associated therapeutic resistance and enhance the effectiveness of cancer treatment.

## Origin of cancer stem cells

**Figure 1.**
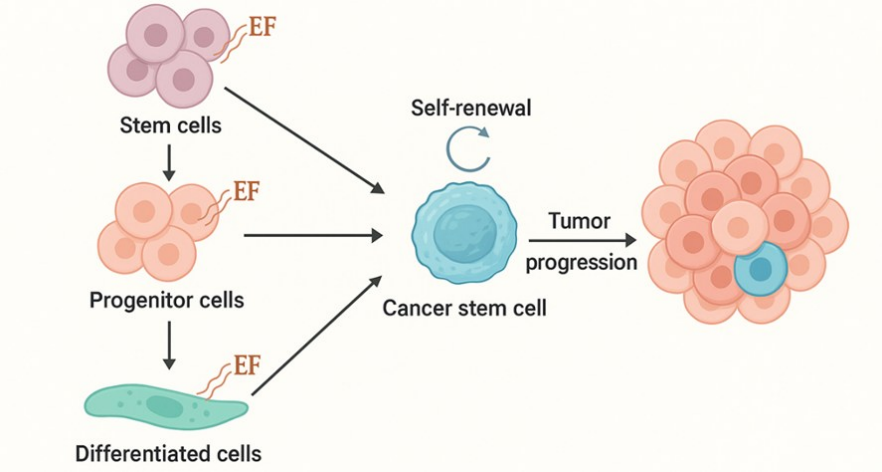
The origins of cancer stem cells (CSCs) are explained through three primary hypotheses: they may arise from normal stem cells, progenitor cells, or differentiated cells that have undergone genetic or epigenetic changes. Each of these pathways suggests distinct mechanisms by which CSCs contribute to tumour initiation, progression, and therapy resistance. Additionally, the role of environmental factors (EF), such as hypoxia, inflammation, and exposure to carcinogens, is crucial in shaping the transformation of these cells into CSCs

Traditionally, cancerous tumours were considered homogeneous masses composed of rapidly dividing cells. However, it is now well-established that tumours are highly heterogeneous and consist of diverse cellular populations. Among these, CSCs play a central role in tumour initiation, progression, and resistance to therapy [14]:

1. From normal stem cells: This hypothesis proposes that CSCs originate from tissue-resident normal stem cells. These cells may undergo oncogenic transformation while retaining their self-renewal and differentiation capabilities. In this scenario, CSCs exploit the same signalling pathways used by normal stem cells to sustain uncontrolled proliferation [15].
2. From progenitor cells: Another theory proposes that CSCs arise from progenitor cells. These cells, which are more abundant in adult tissues than stem cells, possess limited self-renewal potential. Oncogenic mutations in these cells could disrupt regulatory mechanisms, giving rise to CSCs [16].
3. From differentiated cells: The third hypothesis suggests that CSCs can develop from mature, differentiated cells through a process known as dedifferentiation. According to this theory, cancer-causing genetic mutations (oncogenes) trigger the dedifferentiation of mature cells, allowing them to regain stem cell-like properties. This process often involves epithelial-to-mesenchymal transition (EMT), allowing differentiated epithelial cells to acquire mesenchymal and stemness features, ultimately leading to CSC formation [17]. There are three main hypotheses regarding the origin of CSCs, as illustrated in Figure 1.

## Isolation and identification of cancer stem cells

CSCs represent a small fraction of the tumour cell population, typically comprising only 0.01 to 2.00 % of the total tumour mass. This rarity poses significant challenges for their isolation and characterization. CSCs are identified using distinct biomarkers that vary among tumour types, including both solid tumours (*e.g.* prostate, lung, ovarian, liver, brain) and haematological malignancies (*e.g.* chronic myeloid leukaemia). A wide range of cell surface markers has been employed for CSC isolation, including CD44, CD24, CD29, CD90, CD133, CD13, CD271, SOX9, ATP-binding cassette (ABC) transporters (ABCB1, ABCB5, ABCG2), EpCAM, and ALDH1. The biomarkers of CSCs in different cancers are summarized in Table 1 [18].

**Table 1.** Biomarkers of cancer stem cells in different cancers

Types of CSCs origin	Specific marker	Ref.
Breast cancer	CD24, CD44, CD133, CD13, CD14, CD15, CXCR4, EpCAM, LGR5, CD49f, CD90, CD117, CD61, PCOR, ABCG2	[22]
Ovarian cancer	CD133, CD44, CD24, CD117, ALDH1, CD105, CD106, SOX2, EpCAM, ABCB1	[23]
Lung cancer	CD133, CD44, ABCG2, ALDH1A1, EpCAM, Y-box 2, OCT4	[24]
Pancreatic cancer	CD133, CD24, CD44, ESA, Oct4 CXCR4, DCLK1, c-MET, ABCG2, Lgr5, ABCB1	[25]
Colorectal cancer	CD44+, CD144, CD133+, CD26+, CD24, CD29, ALDH1+, CD166+, LGR5, (EpCAM)high, CXCR4	[26]
Prostate cancer	SCA-1, ALDH, CD133, TROP-2, CD44, integrin α2β1, OCT4, NANOG, SOX2	[27]
Brain cancer	CD133, CD44, CD15, CD105, CD70, CD24, Integrin α6, S100A4, ALDH1A3, Nanog, OCT-4, SOX-2, Nestin	[28]
Chronic myeloid leukaemia	IL1RAP, CD93, CD26, CD25	[29]
Melanoma	CD20+, CD133+, ABCB5, ABCB1, ABCG2, CD271+, CD34, CD44	[30]
Hepatocellular carcinoma	CD90, EpCAM, CD133, CD24, SOX9, CK19, SOX12, CD44, CD13, CD47, Lgr5, DLK1, α2δ1, ICAM-1, OV6	[31]

Among these, CD44 is widely recognized for its high expression in CSCs from multiple tumour types. CD44 is a cell surface glycoprotein encoded by the CD44 gene, involved in cell adhesion, migration, and signalling. In haematological cancers, elevated CD44 levels correlate with chemoresistance and poor prognosis.[19]. CD133, also known as prominin-1, is another well-established CSC marker found in tumours of the breast, ovary, pancreas, liver, lung, stomach, and colon [20]. High CD133 expression is associated with enhanced tumorigenic capacity, particularly in lung CSCs, where it contributes to self-renewal, metastasis via EMT, and tumour progression [21].

CD24 is another marker frequently expressed in cancers such as breast, liver, bladder, lung, and ovarian cancer. This marker is critical in promoting tumour development, invasion, metastasis, and apoptosis inhibition, particularly in ovarian cancer [32].

EpCAM is abundantly expressed in colorectal and hepatocellular CSCs, where it facilitates tumour proliferation, invasion, and metastatic spread [33].

In addition to surface markers, intracellular enzymes and membrane transporters also serve as critical indicators of CSC identity and function. One such marker is Aldehyde Dehydrogenase (ALDH), a detoxifying enzyme that contributes to cellular redox balance, signalling pathways, and therapy resistance. In particular, ALDH activity is elevated in prostate CSCs, where it facilitates survival under oxidative stress and drug pressure [34]. In breast CSCs, overexpression of ALDH1A3 is strongly correlated with higher tumour grade, advanced cancer stage, and increased metastatic potential [35].

Another major class of markers includes ABC transporters, which are closely linked to multidrug resistance (MDR). Members such as ABCB1 (MDR1), ABCC1 (MRP1), and ABCG2 (BCRP) are frequently overexpressed in drug-resistant tumours and CSC subpopulations. ABCC1 plays a key role in transporting chemotherapeutic agents like anthracyclines and mitoxantrone, and is found in CSCs of lung, stomach, colon, breast, prostate, neuroblastoma, glioma, and leukaemia [36]. Among these, ABCG2 stands out as a pivotal multidrug-resistant transporter, contributing not only to chemoresistance but also to tumour progression [37]. Its high expression has been reported in a wide range of cancers, including lung, liver, melanoma, and retinoblastoma [38].

Despite the usefulness of surface and intracellular markers in identifying CSCs, several limitations and challenges persist. Many of these markers, such as CD44, CD133, and ALDH, are not exclusively expressed by CSCs but are also found in normal stem or progenitor cells, leading to potential off-target effects and reduced specificity in therapeutic targeting [9]. Moreover, the expression of CSC markers can be highly heterogeneous and dynamic, varying not only among different tumour types but also within a single tumour due to microenvironmental influences and clonal evolution. This heterogeneity complicates the development of universal CSC-targeted therapies and necessitates the use of multiple markers or functional assays for accurate CSC isolation and characterization [39]. Furthermore, some CSC subpopulations may evade detection altogether due to low or transient marker expression, posing a significant obstacle in both research and clinical applications [40]. However, nanostructures offer promising solutions to overcome these limitations in conventional CSCs marker detection.

## Mechanism of resistance to therapeutics in cancer stem cells

**Figure 2.**
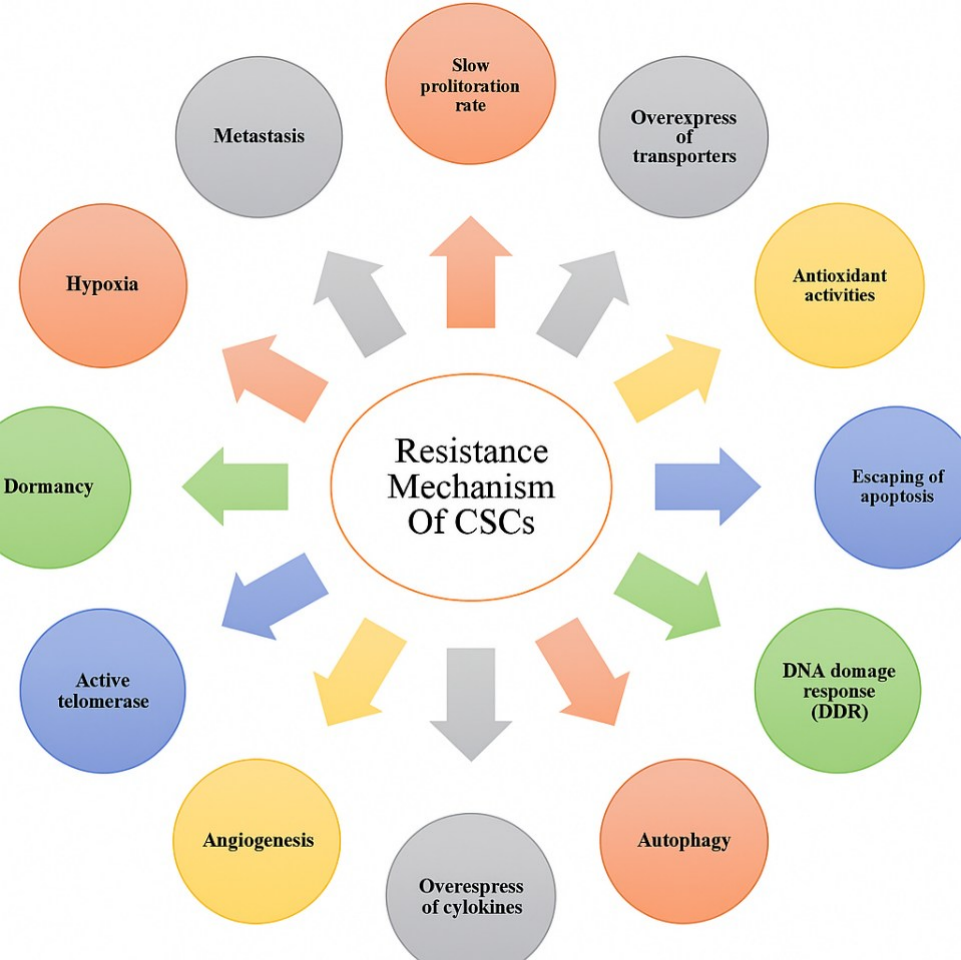
Cancer stem cells employ diverse mechanisms to evade therapeutic interventions, contributing to treatment resistance and disease recurrence.

One of the major obstacles in effective cancer treatment is the inherent resistance of CSCs to conventional therapies, which often leads to disease recurrence. Standard treatments, such as chemotherapy and radiation, typically fail to eradicate CSCs [41]. Several factors contribute to CSCs' resistance, as illustrated in Figure 2.

### Slow proliferation rate

Research indicates that cells with slow cell cycle progression are less susceptible to chemotherapy [42]. Most CSCs reside in the G0 phase, a quiescent state that allows them to evade therapies targeting actively dividing cells. After chemotherapy or radiation, these cells can exit the G0 phase, contributing to cancer recurrence. Conventional therapies mainly target proliferating cells by causing DNA damage and inhibiting mitosis. However, these treatments are less effective against slow-cycling cells like CSCs. Consequently, while chemotherapy may eliminate the bulk of the tumour, CSCs survive and can re-initiate metastasis, leading to relapse [43].

### Overexpression of transporters

ABC transporters are a superfamily of membrane proteins that utilize energy derived from ATP hydrolysis to transport a wide variety of substrates across cellular membranes. These substrates include vitamins, proteins, peptides, amino acids, polysaccharides, drugs, and metal ions [44]. Overexpression of these transporters has been reported in many cancers, particularly in CSCs [45]. By overexpressing ABC transporters, CSCs increase the efflux of chemotherapy drugs, leading to drug resistance [46].

Certain ABC transporters are more highly expressed in CSCs compared to regular cancer cells or normal cells. For example, ABCB1 is overexpressed in pancreatic CSCs, ABCG2 in breast CSCs, ABCB1 and ABCB5 in melanoma, and ABCB1 in ovarian CSCs [47]. Some transporters can transport a wide variety of drugs, while others are more selective. For example, the ABCC1 transporter, overexpressed in glioblastoma CSCs, is involved in the efflux of multiple drugs, including doxorubicin (DOX), methotrexate, paclitaxel (PTX), and others. In contrast, ABCA1, expressed in ovarian CSCs, has a more limited drug transport capacity, primarily transporting cisplatin [48].

### Antioxidant activities

The ALDH superfamily plays a pivotal role in detoxifying endogenous and exogenous aldehydes by catalysing their oxidation into carboxylic acids. These enzymes are highly expressed in CSCs across various cancers, with elevated levels frequently observed in cancer patients. Several ALDH isoforms, including ALDH1A2, ALDH1A3, ALDH1B1, ALDH2, and ALDH3A1, are overexpressed in CSCs [49]. The expression and activity of these enzymes, alongside other biomarkers, have been widely used to identify CSCs in different cancer types. In essence, ALDH enzymes contribute to CSCs' resistance to chemical and oxidative stress [50].

Moreover, CSCs counteract reactive oxygen species (ROS) by upregulating glutathione synthesis, a tripeptide composed of glycine, cysteine, and glutamic acid. Glutathione protects essential cellular components from oxidative damage induced by free radicals, peroxides, and heavy metals [51]. Studies have demonstrated that elevated glutathione levels in CSCs enhance cell viability, promote cell cycle progression, and increase resistance to chemotherapy. Conversely, inhibition of glutathione synthesis impairs CSC self-renewal and sensitizes them to chemotherapeutic agents [52].

### Escaping apoptosis

Apoptosis, a fundamental process for maintaining cellular homeostasis, is tightly regulated, and its disruption is frequently associated with cancer progression. CSCs exhibit significant resistance to apoptosis, contributing to treatment failure and tumour recurrence. For instance, CSCs in human gliomas and leukaemia display reduced expression of Fas and its ligand (FasL), crucial components of the extrinsic apoptotic pathway. Fas, a membrane-bound receptor from the death receptor family, typically initiates cell death upon ligand binding. Reduced expression of Fas and FasL in CSCs leads to diminished sensitivity to apoptotic signals through the extrinsic pathway, thereby enabling CSCs to evade programmed cell death. Moreover, CSCs overexpress inhibitors of apoptosis proteins (IAPs), such as XIAP, c-IAP1, and Livin, which act as potent suppressors of the intrinsic apoptotic pathway. These proteins inhibit the activation of caspases — the central executioners of apoptosis-thus further enhancing the survival of CSCs under stress conditions that would typically eliminate non-stem cancer cells [53].

Another crucial mechanism contributing to apoptosis resistance in CSCs is the activation of the G2/M phase cell cycle checkpoint. This checkpoint is triggered in response to DNA damage or other cellular stressors, halting the cell cycle before mitosis. In CSCs, this leads to a prolonged G2/M arrest, allowing time for DNA repair and promoting survival. This selective advantage not only confers resistance to chemotherapy and radiation, which primarily target rapidly dividing cells, but also enhances tumorigenic potential, as cells in the G2/M phase are often more resistant to apoptosis-inducing agents [54]. This complex network of apoptosis evasion strategies highlights the major challenge in targeting CSCs during cancer treatment. Their ability to resist both intrinsic and extrinsic apoptotic signals, coupled with sustained G2/M checkpoint activation, enables them to survive under hostile therapeutic conditions, contributing to tumour persistence and recurrence. A deeper understanding of these mechanisms could facilitate the development of more effective therapies specifically designed to target CSCs and overcome their apoptotic resistance [55].

### DNA damage response

A key factor contributing to the survival of CSCs under stressful conditions, such as chemotherapy and radiation, is their enhanced capacity for DNA repair [56]. This DNA damage response (DDR) is crucial for CSCs to maintain genomic integrity, as it enables them to survive therapies that induce DNA damage, a common means of killing cancer cells. The ability to repair DNA following such treatments not only protects CSCs from apoptosis but also preserves their tumorigenic potential [55].

One of the primary protective mechanisms in CSCs is their ability to alleviate oxidative stress by scavenging ROS, which are major contributors to DNA damage. CSCs, particularly those found in breast cancer, have been shown to maintain lower intracellular ROS levels, thereby minimizing oxidative DNA injury. In addition, CSCs enhance their DNA repair capabilities by activating critical components of the DDR. For instance, the phosphorylation of ataxia-telangiectasia mutated (ATM) and checkpoint kinases (CHK1/CHK2) is pivotal in promoting efficient DNA repair and cell cycle regulation. These proteins are central to recognizing DNA damage and halting the cell cycle, allowing time for repair, which ultimately prevents apoptosis [57].

Moreover, CSCs are adept at activating anti-apoptotic signalling pathways, such as Notch, PI3K/Akt, and WNT/β-catenin, which not only enhance their survival but also bolster their ability to repair single-strand breaks in DNA. Research has shown explicitly that breast CSCs have a more active single-strand break repair pathway, contributing to their resilience against therapeutic interventions. This robust repair mechanism further reinforces the survival of CSCs by reducing the likelihood of treatment-induced cell death, thereby contributing to resistance against chemotherapy and radiation [58].

In gliomas, CD133^+^ CSCs have been identified as key contributors to radioresistance and tumour regeneration. These glioma stem cells display enhanced checkpoint activation and more efficient DNA repair, allowing them to withstand radiation-induced damage and recover more effectively than non-stem tumour cells. By rapidly repairing DNA lesions, these CSCs not only evade apoptosis but also drive tumour recurrence, making them critical targets for therapeutic interventions aimed at minimizing relapse [59].

Indeed, the superior DNA repair mechanisms observed in CSCs provide a substantial survival advantage, enabling them to resist conventional treatments such as chemotherapy and radiation. This ability, combined with efficient ROS detoxification and activation of survival signalling, highlights the resilience of CSCs and emphasizes the urgent need for novel therapeutic strategies that specifically target their DNA repair machinery [60].

### Autophagy

Autophagy plays a critical role in promoting the resistance of CSCs to chemotherapy and radiation therapy. This cellular process functions as a survival mechanism, enabling cells to adapt to various stressors, including nutrient deprivation, hypoxia, and therapeutic interventions. During autophagy, damaged or redundant cellular components are degraded and recycled into essential metabolites, which are then utilized to support cell survival under adverse conditions [61].

In CSCs, autophagy not only enables adaptation to the harsh TME but also contributes directly to therapeutic resistance. By recycling intracellular components, autophagy provides energy and biosynthetic precursors necessary for cellular repair and survival, especially when external resources are scarce. Moreover, studies suggest that inhibiting autophagy can induce apoptosis in cancer cells, indicating that autophagy serves as a protective shield against cell death pathways [61].

Numerous studies have documented the significant role of autophagy in various types of cancers, including pancreatic cancer, bladder cancer, colorectal cancer, chronic myeloid leukaemia, and glioblastoma [62]. In these cancers, autophagy contributes to tumour cell survival, therapy resistance, and disease progression. In CSCs, in particular, autophagic activity is often elevated, as evidenced by the overexpression of key autophagic markers such as ATG5 and Beclin1, which reflect increased autophagic flux [61]. This heightened autophagy enables CSCs to maintain their metabolic needs and protect themselves from the cytotoxic effects of treatments aimed at inducing oxidative stress and DNA damage [63].

Furthermore, autophagy in CSCs plays an essential role in regulating intracellular ROS levels, which typically increase during cancer treatments. Through protective autophagic pathways, CSCs maintain low ROS levels, thereby preventing oxidative damage that could otherwise trigger cell death. This capacity to modulate oxidative stress is a key contributor to their resistance to therapies relying on ROS-mediated cytotoxicity.

Overall, autophagy represents a double-edged sword in CSC biology. While it enhances survival and resistance to standard therapies, it also offers a promising therapeutic target. Inhibiting autophagy in CSCs may sensitize them to oxidative stress and apoptosis, potentially improving the effectiveness of treatments such as chemotherapy and radiation. A deeper understanding of autophagic processes in CSCs could pave the way for more effective strategies to overcome resistance and prevent tumour recurrence [63].

### Overexpression of cytokines

There is substantial clinical and experimental evidence linking chronic inflammation to cancer development, with growing research emphasizing the role of CSCs in this process. CSCs can secrete specific cytokines that not only modulate the TME but also enhance their resistance to chemotherapeutic agents. For example, in breast cancer, cytokines present in the TME have been shown to regulate CSC self-renewal, survival, and contribute to therapy resistance through multiple mechanisms [64]. Inflammatory cytokines, such as interleukin-1 (IL-1), interleukin-6 (IL-6), and interleukin-8 (IL-8), have been implicated in tumour progression by influencing the CSC population.

IL-1 levels are often elevated in patients with metastatic cancer, suggesting a pro-tumorigenic role in promoting aggressive cancer phenotypes. Similarly, IL-8 plays a pivotal role in breast cancer, where increased expression of IL-8 by CSCs enhances their self-renewal capacity and accelerates tumour growth. The ability of these inflammatory cytokines to sustain CSC survival and proliferation underscores the complex interplay between inflammation and cancer. In this context, the inflammatory microenvironment not only promotes tumour progression but also reinforces CSC-mediated resistance to conventional therapies [65].

Targeting cytokine-driven signalling pathways thus represents a promising therapeutic approach to reduce CSC-associated chemoresistance and tumour recurrence. Elucidating the roles of key inflammatory cytokines in modulating CSC dynamics may offer new avenues for the development of more effective treatments, particularly for aggressive and treatment-resistant cancers.

### Angiogenesis

Angiogenesis, the process of forming new blood vessels, is essential for tumour development, as it allows tumours to establish a vascular network that supplies oxygen and nutrients while facilitating the removal of waste products. This vascularization is critical for tumour growth and survival. Although angiogenesis plays a vital role in normal physiological processes, it is also a hallmark of various pathological conditions, including cancer. In tumours, the self-renewal capacity of CSCs and the initiation of tumorigenesis are closely associated with the overexpression of key angiogenic markers, such as vascular endothelial growth factor (VEGF), VEGF receptors (VEGFR1 and VEGFR2), and hypoxia-inducible factors (HIF-1α and HIF-2α). These molecules drive the angiogenic cascade, which in turn supports the expansion and maintenance of the CSC population [66].

The role of angiogenesis in supporting CSCs is particularly significant, as the enhanced blood supply ensures CSC viability and facilitates their proliferation. By improving nutrient and oxygen delivery, angiogenesis creates a microenvironment conducive to CSC maintenance and tumour progression. Moreover, studies have shown that in gliomas, CSCs actively participate in angiogenesis by secreting pro-angiogenic factors, such as stromal-derived factor 1 (SDF-1) and VEGF, thereby promoting the formation of new tumour blood vessels [67]. This not only sustains tumour growth but also protects CSCs from therapeutic stress, reinforcing their resistance through the formation of a supportive vascular niche.

Indeed, the interplay between CSCs and angiogenesis represents a critical therapeutic target. Disrupting angiogenic signalling pathways, particularly those mediated by VEGF and related factors, may compromise CSC survival and reduce tumour vascularization. Understanding the reciprocal relationship between CSCs and angiogenesis provides valuable insights for developing novel therapeutic strategies aimed at limiting tumour progression and overcoming treatment resistance.

### Active telomerase

Telomerase is an RNA-dependent DNA polymerase enzyme that plays a critical role in maintaining telomere length, which is essential for chromosomal stability [68]. While telomerase remains inactive in most normal somatic cells, its reactivation is observed in nearly 90 % of human cancers. In CSCs, telomerase activity is markedly elevated and is crucial for maintaining their self-renewal capacity, survival, and immortality. This upregulated activity allows CSCs to bypass replicative senescence, thereby supporting the uncontrolled proliferation and persistence of tumours [69].

A key component of telomerase is the human telomerase reverse transcriptase (hTERT), the catalytic subunit responsible for adding telomeric repeats to chromosome ends. Studies have demonstrated that hTERT overexpression in CSCs significantly increases their population, as seen in gastric cancer. This indicates that telomerase is not only essential for telomere maintenance but also directly contributes to CSC proliferation and tumorigenesis [70].

Moreover, impaired telomere maintenance can severely compromise CSC self-renewal. In the absence of adequate telomerase activity, telomere shortening may trigger cellular senescence or apoptosis, limiting the tumorigenic potential of CSCs. Thus, telomerase plays a pivotal role in ensuring the longevity and sustainability of CSC populations within tumours [71].

Given its fundamental role in CSC biology, telomerase is considered a promising therapeutic target. Inhibiting telomerase activity in CSCs could potentially abrogate their immortality, thereby reducing tumour growth and recurrence. Targeting telomerase may offer a powerful strategy for eliminating treatment-resistant cancer cells. Understanding the regulatory mechanisms of telomerase expression and activity in CSCs is essential for the development of effective and durable anti-cancer therapies.

### Dormancy

The concept of cancer cell dormancy was first introduced by Rupert Willis in 1934 [72], when he observed cases of late metastasis in patients without any evidence of local recurrence. He hypothesized that cancer cells had migrated to secondary tissues and "must have lain dormant" for extended periods. Dormancy is now recognized as an adaptive survival mechanism employed by cancer stem cells to evade unfavourable microenvironmental conditions, including chemotherapy, radiation, nutrient deprivation, and other stressors. In their dormant state, CSCs remain viable but temporarily suspend proliferation, enabling them to survive under harsh conditions and escape detection by conventional therapies [73].

**Figure 3.**
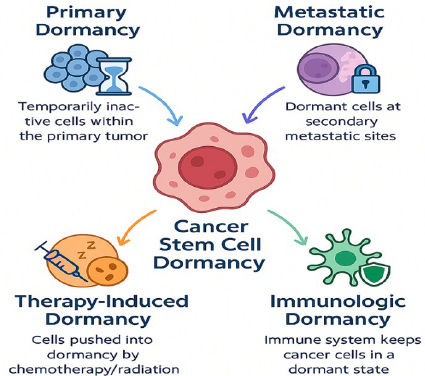
Different types of cancer stem cell dormancy. Therapy-induced dormancy occurs as a survival strategy following chemotherapy or radiotherapy. Metastatic dormancy refers to the quiescent state of disseminated cancer stem cells at secondary sites. Primary dormancy represents the intrinsic quiescence of cancer stem cells within the primary tumour niche. Immunological dormancy is maintained through immune surveillance and tumour-immune system interactions

There are several types of CSCs dormancy, each contributing to the complexity of cancer progression and recurrence: 1. Primary cancer dormancy: Cancer cells within the primary tumour temporarily cease proliferation but remain viable, ready to resume growth when environmental conditions become favourable, 2. Metastatic dormancy: Disseminated tumour cells (DTCs) that have spread to distant organs enter a dormant state, sometimes for years or even decades, before reactivating to initiate metastatic growth, 3. Therapy-induced dormancy: Exposure to chemotherapy or radiation may drive CSCs into a quiescent state, allowing them to survive treatment and later re-emerge, potentially leading to tumour recurrence, 4. Immunologic dormancy: CSCs remain non-proliferative due to constant immune surveillance, which prevents their expansion without fully eliminating them [74]. The types of cancer dormancy are shown in Figure 3.

Understanding the mechanisms of dormancy is crucial for developing effective long-term cancer therapies, as dormant CSCs are believed to be a key source of resistance, relapse, and metastasis.

The dormancy phenomenon poses a significant challenge to cancer treatment. Dormant CSCs are particularly problematic because they can evade therapies targeting rapidly dividing cells. Furthermore, different treatments can have varying effects on dormant cells. Some therapies may induce dormancy, thereby inhibit metastasis and slow disease progression. Others, however, can inadvertently activate dormant CSCs, leading to tumour recurrence and the development of more aggressive forms of cancer [75].

This duality has sparked debate among researchers about the best therapeutic approach. Some advocate for dormancy induction as a useful strategy, believing it can prevent metastasis and maintain the cancer in a controlled, non-progressive state. On the other hand, others argue that activating dormant CSCs followed by their targeted elimination may be more effective, as it could eradicate the hidden reservoir of cancer cells that fuel recurrence. Understanding the mechanisms governing dormancy in CSCs is essential for developing more effective treatment strategies. Whether inducing or disrupting dormancy proves to be the better approach will depend on deeper insights into how these cells respond to different therapies and how dormancy contributes to long-term cancer survival and relapse.

### Hypoxia

Low oxygen levels, or hypoxia, play a critical role in maintaining CSCs and promoting their malignancy, thereby contributing to tumour survival, progression, and metastasis. Under hypoxic conditions, the expression of hypoxia-inducible factors (HIFs), particularly HIF-1α and HIF-2α, is significantly upregulated. These transcription factors are essential regulators of cellular adaptation to low oxygen levels and are strongly associated with tumour aggressiveness, metastasis, and angiogenesis induction. Elevated HIF expression correlates with increased malignancy and resistance to conventional therapies such as chemotherapy and radiotherapy [76].

For example, Glioblastoma multiforme (GBM), the most aggressive form of brain cancer, is characterized by extensive hypoxic regions. Glioblastoma stem cells (GSCs) preferentially inhabit these hypoxic niches, exhibiting enhanced self-renewal, proliferation, and invasive potential. Evidence suggests that hypoxia is a major driver of GSC malignancy and their resistance to chemo- and radiotherapy. This hypoxia-mediated resistance poses a significant challenge in GBM treatment, as GSCs can survive and adapt under conditions that are lethal to non-stem cancer cells [77].

Moreover, hypoxia has been shown to contribute to drug resistance in tumours, primarily through the regulation of drug transporters. A strong correlation between HIF-1α and the expression of drug transporters, such as ABCB1 (also known as MDR1), has been observed. ABCB1 is involved in the efflux of chemotherapeutic agents from cancer cells, contributing to multidrug resistance [78]. The HIF-1α binding site in the promoter region of the MDR1 gene has been identified, demonstrating that HIF-1α can directly upregulate the expression of this transporter. This interaction underscores the role of hypoxia in driving not only CSCs' survival and malignancy but also their ability to resist multiple drugs, making treatment more challenging.

In summary, understanding the impact of hypoxia on CSCs and tumour biology underscores the necessity of developing therapeutic approaches targeting HIF signalling pathways and the hypoxic TME. Strategies that inhibit HIF activity or improve tumour oxygenation may reduce CSC-mediated drug resistance and improve the effectiveness of existing cancer treatments.

### Metastasis

Metastasis is a defining hallmark of malignant tumours and a major determinant of cancer patient prognosis and survival. It is a complex, multistep cascade through which cancer cells disseminate from the primary tumour and establish secondary tumours at distant sites. A pivotal event in this cascade is the epithelial-to-mesenchymal transition (EMT), a phenotypic shift whereby epithelial tumour cells lose their polarity and cell-cell adhesion properties and acquire a more motile and invasive mesenchymal phenotype. This transition enhances cellular plasticity, increases metabolic activity, and facilitates migration and invasion, all of which are crucial for tumour spread [79].

In normal epithelial tissues, strong intercellular adhesion, maintained by tight and adhesive junctions, preserves structural integrity and limits cell motility. However, during EMT, the disruption of these junctions enables tumour cells to detach from the primary site. As epithelial traits are lost, cells adopt a spindle-like morphology and acquire mesenchymal features, allowing them to infiltrate surrounding tissues and gain access to the circulatory or lymphatic systems, thereby initiating metastasis [80].

CSCs play a crucial role in the initiation and progression of metastasis. Studies have shown that CSCs express Tenascin C (TNC), an extracellular matrix glycoprotein involved in tissue remodelling and metastasis. The ability of CSCs to produce TNC has been strongly linked to metastasis, particularly in lung cancer models. Experimental evidence suggests that TNC expression by CSCs significantly contributes to the metastatic process, as reducing TNC levels in breast cancer cells decreases their migratory capacity, highlighting its role in cell migration and tumour dissemination [81]. CSCs have been recognized as key mediators of metastasis across multiple tumour types, including brain, breast, uterine, ovarian, and pulmonary cancers. These cells often exhibit remarkable plasticity and adaptability, which enable them to endure the hostile microenvironments of distant organs and initiate secondary tumour formation. The strong association between CSCs and metastasis highlights the urgent need for therapies specifically targeting the metastatic capabilities of these cells [82].

Targeting CSC-mediated metastasis offers promising therapeutic opportunities. Potential strategies include inhibiting EMT, downregulating TNC expression, or disrupting the metastatic niche. By effectively suppressing the metastatic mechanisms driven by CSCs, such approaches could improve treatment outcomes and reduce cancer recurrence.

## Cancer treatment methods and their limitations

Chemotherapy and radiotherapy remain two of the most widely utilized treatments for cancer. However, these conventional approaches often demonstrate limited efficacy and are accompanied by substantial side effects. While they may effectively destroy bulk tumour cells, they typically fail to eradicate CSCs, which are inherently more resistant to such treatments. CSC resistance is attributed to mechanisms such as enhanced DNA repair capacity, increased drug efflux activity, and activation of anti-apoptotic pathways. This therapeutic shortcoming significantly compromises the overall success of chemotherapy and radiotherapy.

Surgical intervention is another standard treatment modality, yet it is not devoid of complications. Post-operative issues, including infections, can trigger inflammatory responses that may inadvertently promote tumour recurrence. This is largely mediated by the release of pro-inflammatory cytokines and growth factors that enhance cancer cell survival and proliferation [83].

The advent of cancer immunotherapy in 2013 marked a transformative milestone in oncology. This approach leverages the patient’s immune system to recognize and eliminate cancer cells, offering new perspectives in tumour immunology, particularly in the identification of tumour-specific antigens and the development of targeted immunotherapeutic agents [84]. Despite its revolutionary potential, immunotherapy presents notable challenges, including autoimmune reactions, variable clinical responses, and severe adverse events such as cytokine storms, all of which necessitate further refinement and personalized application [85].

Hormone therapy represents an additional strategy, particularly effective for hormone-dependent malignancies such as breast and prostate cancers. It can be administered preoperatively to reduce tumour size or as an adjunct to radiotherapy [86]. However, in breast cancer patients, hormone therapy frequently induces a spectrum of undesirable side effects, including cognitive dysfunction, fatigue, hot flashes, anxiety, insomnia, pain, and weight gain [87].

Photodynamic therapy (PDT) is a non-invasive treatment that has shown particular efficacy in cutaneous malignancies and superficial epithelial tumours. PDT may also be used synergistically with chemotherapy, radiotherapy, or immunotherapy to improve therapeutic outcomes [88].

Gene therapy offers another promising frontier in cancer treatment. This technique involves the delivery of genetic material, DNA or RNA, into host cells via vectors, with the aim of either correcting genetic defects or silencing oncogenic pathways. By restoring the expression of therapeutic genes or inhibiting tumour-promoting genes, gene therapy holds the potential to significantly enhance clinical outcomes [89].

Despite these advances, most of these treatment modalities are largely ineffective against CSCs, which are often implicated in tumour relapse, metastasis, and resistance to therapy. In this context, nanotechnology emerges as a powerful and versatile platform that enables the development of innovative strategies to specifically target and eliminate CSCs. This technology holds great promise in overcoming the limitations of conventional therapies and advancing the efficacy of cancer treatment.

## Application of nano-biomaterials in targeting cancer stem cells

Cancer stem cells (CSCs) constitute a small, yet critical subpopulation of tumour cells present in both solid and haematological malignancies. These cells play a pivotal role in therapy resistance, metastasis, and disease relapse. Due to their unique properties, including self-renewal and differentiation into diverse tumour cell types, CSCs often exhibit resistance to conventional cancer treatments, making them an essential target for innovative therapeutic strategies [90]. Therefore, there is an urgent need for effective therapeutic strategies that can selectively target CSCs, which highlights the difference between conventional therapies and approaches specifically designed to eradicate these cells.

**Figure 4.**
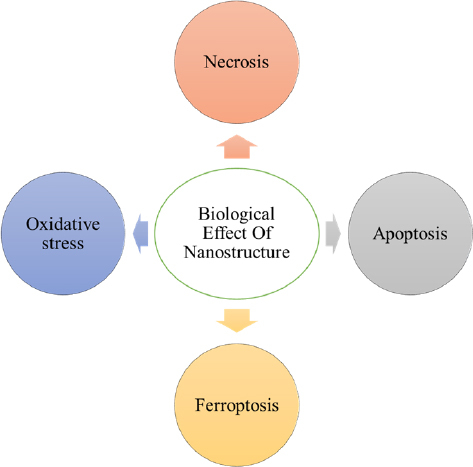
Biological effects of nanostructures on cancer stem cell physiology.

In recent years, nanotechnology has emerged as a promising platform for targeting CSCs, providing novel diagnostic and therapeutic opportunities. Various nanomaterials, including metallic and metal-oxide based nanoparticles, carbon-based nanomaterials, and organic nanoparticles have demonstrated potential in overcoming CSC-associated challenges [91]. The unique physicochemical properties of these nanomaterials allow them to circumvent limitations of traditional therapies, such as poor drug solubility, non-specific biodistribution, and limited bioavailability. Furthermore, by specifically targeting CSCs, nanomaterials hold promise to reduce metastasis and cancer recurrence, thereby improving patient outcomes. Several nanomaterials have exhibited efficacy in preclinical models for CSC targeting [92]. These materials, along with their applications in both diagnostics and therapeutics, are summarized in Table 2. Also, the biological effects of nanostructures on cell physiology are shown in Figure 4.

**Table 2.** Types of nanostructures in targeting cancer stem cells.

Types of CSCs origin	Type of nanostructure	The biological effect of nanostructure	Ref.
Breast cancer	Au nanoparticles coated with poly (ethylene glycol), loaded with salinomycin	Induce ferroptosis and Oxidative stress	[93]
Head and neck cancer	Ag nanoparticles	Induce apoptosis by stopping the cell cycle	[94]
Pancreatic cancer	CuO nanoparticles	Induced apoptosis by producing ROS	[95]
Breast cancer	ZnO nanoparticles	Induce apoptosis	[96]
Quiescent colorectal CSCs	Iron oxide nanoparticles coated with heat-sensitive polymer (Poly(DEGMA-co-PEGMA)), loaded with doxorubicin	Magnetic hyperthermia in combination with local chemotherapy as a dual treatment inhibits dormant CSCs	[97]
Ovarian cancer	SPIONs	Induce oxidative stress, decrease autophagy activity, activate ferroptosis, inhibit proliferation and invasion, decrease drug resistance and tumorigenic ability by producing ROS	8
Hominid testicular embryonic	Ag nanoparticles synthesized using green tea (Camellia sinensis)	Ag nanoparticles increase the expression of Bcl2, Bax, caspase 3 and 9, p53 and HSPA2 genes and are considered as an apoptosis-inducing complex	[99]
Breast cancer	Carbon nanotubes	Hyperthermia with carbon nanotubes causes more membrane permeability and breast CSCs necrosis	[100]
Ovarian cancer	GO-Ag nanocomposite	Generation of ROS, leakage of lactate dehydrogenase, reduction of mitochondrial membrane potential, and enhanced expression of apoptotic genes	[101]
Breast cancer	Multi-walled carbon nanotubes (MWCNTs) with Fe_3_O_4_ nanoparticles on the inner and Au nanoparticles on the outer surface, combined with paclitaxel	Induced apoptosis	[102]
Breast cancer	pH-sensitive liposomes loaded with bufalin and doxorubicin	Inhibited tumorigenesis and self-renewal	[103]
Breast cancer	Lipid nano capsule containing the drugs salinomycin and paclitaxel	Reduced tumour growth and induced apoptosis	[104]
Pancreatic cancer	Nano capsules loaded with paclitaxel	Anti-tumour effectiveness of the drug encapsulated in the nano capsule was four times higher than that of the free drug	[105]
Cancer stem-like cells	Chitosan-decorated doxorubicin-encapsulated	Increases the cytotoxicity by six times	[106]
Pancreatic cancer	PLGA loaded with anthothecol	Prevented cell proliferation and colony formation and induced apoptosis	[107]

### Metal-based nanomaterials

Metal-based nanoparticles, such as copper (Cu), gold (Au), silver (Ag), titanium dioxide (TiO_2_), iron oxide (Fe_3_O_4_), manganese oxide (Mn_3_O_4_), zinc oxide (ZnO), and magnesium oxide (MgO), have gained widespread application across diverse fields including industry, biomedicine, wastewater treatment, pharmaceuticals, cosmetics, catalysis, and energy storage [108-111]. In biomedicine, they play a prominent role in disease diagnosis (*e.g.* imaging techniques) and therapy (e.g., targeted drug delivery, photothermal therapy, and hyperthermia) [112-114].

This section highlights the application of metal-based nanoparticles in detecting and eliminating CSCs. Functionalized metallic nanoparticles, particularly magnetic and gold-based, can be conjugated with specific antibodies or ligands that target CSC surface markers such as CD44, CD133, or CD24. These engineered nanostructures enhance CSC detection through improved imaging contrast (*e.g.* MRI, photoacoustic, or fluorescence imaging) or via magnetic separation techniques.

For instance, magnetic nanoparticles functionalized with anti-CD44 antibodies have been successfully used to isolate and enrich CSCs from head and neck squamous cell carcinoma (HNSCC) samples. In this study, a CD24-gold nanocomposite demonstrated enhanced diagnostic and prognostic capabilities in detecting CSCs in salivary gland tumours, outperforming conventional diagnostic methods [115].

Gold nanoparticles have also been employed for therapeutic purposes. Zhao *et al.* [93] conjugated salinomycin, an antibiotic with anti-CSC activity, with PEGylated gold nanoparticles. The resulting nanocomplex selectively targeted breast CSCs, inducing ferroptosis and oxidative stress.

Kaur *et al.* [94] reported that silver nanoparticles could trigger apoptosis in CSCs derived from head and neck cancers by inducing cell cycle arrest. The study, using CSCs from the Cal33 cell line, found that relatively high IC50 values were required, indicating selective cytotoxicity.

Copper oxide (CuO) nanoparticles have also shown promise in CSC inhibition. It was found that they induce apoptosis in pancreatic CSCs through the generation of ROS [95]. Similarly, Fakhroueian *et al.* [96] demonstrated that ZnO nanoparticles effectively induced apoptosis in breast CSCs.

Magnetic nanoparticles provide a novel approach to CSC inhibition via magnetic hyperthermia. Superparamagnetic iron oxide nanoparticles (SPIONs), when exposed to alternating magnetic fields (AMFs), produce localized heating in tumour tissues [116]. The main advantage of magnetic hyperthermia is its controllability, as the heating rate can be fine-tuned by adjusting nanoparticle size, shape and AMF properties. Several preclinical studies have confirmed the potential of magnetic hyperthermia in cancer treatment [117]. For example, Sadhukha *et al.* [118] demonstrated that magnetic hyperthermia using SPIONs more effectively eradicated CSCs in breast and lung cancer models compared to conventional water-bath hyperthermia, resulting in both necrosis and apoptosis.

Further research by Fernandes *et al.* [97] explored combining magnetic hyperthermia with local chemotherapy to target dormant colorectal CSCs. In this study, cubic iron oxide nanoparticles were coated with a heat-sensitive polymer (poly(DEGMA-co-PEGMA)) and loaded with DOX. The polymer released DOX at temperatures above 44 °C, causing dormant CSCs to become active. Once activated, the CSCs absorbed the released DOX, which enhanced the cytotoxic effect on tumour cells.

In addition to magnetic systems, gold nanoparticles have been utilized for photothermal hyperthermia. A study by Xu *et al.* [119] showed that Au nanorods, upon exposure to near-infrared (NIR) light, generated localized heat sufficient to eliminate breast CSCs. The treatment led to reduced expression of stemness-related genes such as ALDH1 and KLF4, suggesting effective stem cell ablation.

### Carbon-based nanomaterials

Carbon-based nanomaterials (CBNs, including fullerenes, carbon nanotubes (CNTs), carbon nanofibers, graphene quantum dots (GQDs), graphene oxide (GO), and nanodiamonds) possess unique physical and chemical properties that make them highly valuable in biomedical applications. These materials are widely used in fields such as targeted drug delivery, biomedical imaging, biosensors, tissue engineering, and cancer therapy, including the inhibition of CSCs [120,121].

Carbon-based nanomaterials have been extensively explored for both the detection and inhibition of CSCs, owing to their unique physicochemical properties and ability to be functionalized with targeting ligands such as CD44 antibodies. For example, in the study by Al Faraj *et al.* [122], PEGylated single-walled carbon nanotubes (SWCNTs) were conjugated with superparamagnetic iron oxide nanoparticles (SPIONs) and further functionalized with anti-CD44 antibodies to specifically target breast CSCs. This multifunctional nanoplatform enabled the dual-modal imaging of CSCs using both near-infrared fluorescence imaging and MRI. The system demonstrated high specificity and accumulation at tumour sites overexpressing CD44, thereby providing a promising approach for the non-invasive detection and monitoring of CSCs in breast cancer.

Also, carbon-based nanomaterials have emerged as promising platforms for the suppression of CSCs. Burke *et al.* [100] utilized carbon nanotube-mediated hyperthermia to target breast CSCs. Their results demonstrated that thermal treatment enhanced membrane permeability and induced necrosis in these cells.

In another study, Choi *et al.* [101] developed a graphene oxide-silver (GO-Ag) nanocomposite that exhibited significant cytotoxicity against ovarian CSCs by increasing ROS generation, inducing lactate dehydrogenase (LDH) leakage, disrupting mitochondrial membrane potential, and upregulating apoptotic gene expression. Notably, its combination with salinomycin enhanced the apoptotic response by fivefold compared to individual treatments.

Ghoderao *et al.* [102] reported the synthesis of magnetic multi-walled carbon nanotubes (MWCNTs), functionalized with iron oxide (Fe_3_O_4_) nanoparticles on the inner surface and gold nanoparticles on the outer surface, and loaded with paclitaxel (PTX). This multifunctional system effectively suppressed breast CSCs and triggered apoptosis.

Likewise, Miao *et al.* [123] synthesized both unmodified (SWCNT-Raw) and modified (SWCNT-COOH) single-walled carbon nanotubes. These nanotubes were found to inhibit the dedifferentiation of osteosarcoma stem cells, reduce tumour growth and micro vessel density, limit the acquisition of stem cell phenotypes, and enhance apoptosis in the osteosarcoma microenvironment.

### Organic nanostructures

Organic nanostructures, including micelles, dendrimers, liposomes, nanogels, polymeric nanoparticles (such as nanospheres, nano capsules, and protein-based nanocarriers), and layered biopolymers, are widely recognized for their excellent biocompatibility and biodegradability [124]. In recent years, these nanostructures have shown significant promise in the detection and targeted elimination of CSCs.

Gao *et al.* [125] developed pH-sensitive liposomes to target breast CSCs using trastuzumab-sensitive and resistant HER2^+^ breast cancer models. Their study showed that combining bufalin and DOX in liposomes reduced the breast CSCs population by 85 % and inhibited both tumorigenesis and self-renewal, though it had little effect on cell migration and invasiveness.

Basu *et al.* [104] used lipid nano capsules co-loaded with salinomycin and paclitaxel (PTX) to target breast CSCs. The simultaneous delivery of these drugs significantly inhibited tumour growth and induced apoptosis.

In addition, Navarro-Marchal *et* al. [105] developed anti-CD44-conjugated olive oil liquid nano capsules to target pancreatic CSCs. Encapsulation of PTX in these nanocarriers resulted in a fourfold increase in antitumor efficacy compared to the free drug.

Rao *et al.* [106] created chitosan-decorated DOX-encapsulated nanoparticles to target and eliminate cancer stem-like cells. These nanoparticles specifically targeted the CD44 receptor, enhancing cytotoxicity sixfold by efficiently releasing the drug. Verma *et al.* [107] utilized PLGA nanoparticles encapsulating anthothecol, an antimalarial agent, to target pancreatic CSCs. This formulation inhibited cell proliferation and colony formation, while inducing apoptosis in both pancreatic CSCs and cancer cell lines, without affecting normal pancreatic ductal epithelial cells.

## Conclusions

Cancer stem cells (CSCs) have emerged as critical players in the pathogenesis, progression, and recurrence of cancer, posing formidable challenges to conventional treatment strategies. Their intrinsic capabilities for self-renewal, differentiation, and therapy resistance make them key drivers of metastasis and treatment failure. The resistance mechanisms employed by CSCs, including slow proliferation rates, enhanced DNA repair, evasion of apoptosis, autophagy, and survival under hypoxic conditions, highlight the complexity of effectively targeting these cells. Moreover, the interaction of CSCs with the TME, facilitated by angiogenesis, inflammatory cytokines, and their role in metastasis, underscores their adaptability and resilience. Nanotechnology has opened transformative avenues in combating CSCs. Metal-based nanoparticles, carbon-based nanomaterials, and organic nanostructures provide novel strategies to overcome the limitations of traditional cancer therapies.

These nanomaterials enhance therapeutic efficacy by enabling precise targeting, improving drug delivery, and disrupting the protective mechanisms of CSCs. Additionally, advances in nanoparticle-mediated hyperthermia and induction of oxidative stress have demonstrated promising preclinical results, highlighting their potential to selectively eliminate CSCs while minimizing damage to normal cells. Despite significant progress, challenges remain in translating these technologies into clinical practice. Issues such as biocompatibility, scalability, and long-term safety require thorough investigation through rigorous research and clinical trials. Furthermore, a deeper understanding of CSC biology and the development of multimodal approaches that combine nanotechnology with other therapeutic modalities could further improve outcomes.

In conclusion, targeting CSCs with innovative nano-biomaterials represents a promising frontier in cancer treatment. By addressing the root causes of therapy resistance and recurrence, these strategies hold the potential to revolutionize cancer care, enhance survival rates, and reduce the burden of metastatic disease. Continued interdisciplinary collaboration and sustained investment in this field will be pivotal in advancing the clinical application of these cutting-edge technologies.

## Abbreviations

ABC: ATP-binding cassette
ABCB1: ATP-binding cassette subfamily B member 1
ABCB5: ATP-binding cassette subfamily B member 5
ABCG2: ATP-binding cassette subfamily G member 2
ALDH: Aldehyde dehydrogenase
ALDH1: Aldehyde dehydrogenase 1
ALDH1A1: Aldehyde dehydrogenase 1 family member A1
ALDH1A3: Aldehyde dehydrogenase 1 family member A3
AMF: Alternating magnetic field
Au: Gold
Ag: Silver
Bcl2: B-cell lymphoma 2
Bax: Bcl-2-associated X protein
CD: Cluster of differentiation
CD13: Aminopeptidase N
CD24: Cluster of differentiation 24
CD44: Cluster of differentiation 44
CD49f: Integrin alpha-6
CD90: Thy-1 cell surface antigen
CD105: Endoglin
CD117: c-Kit receptor tyrosine kinase
CD133: Prominin-1
CK19: Cytokeratin 19
CSC: Cancer stem cell
CSCs: Cancer stem cells
Cu: Copper
CuO: Copper oxide
CXCR4: C-X-C chemokine receptor type 4
DCLK1: Doublecortin-like kinase 1
DLK1: Delta-like non-canonical Notch ligand 1
DNA: Deoxyribonucleic acid
DOX: Doxorubicin
EMT: Epithelial-to-mesenchymal transition
EpCAM: Epithelial cell adhesion molecule
ESA: Epithelial-specific antigen
EF: Environmental factors
Fe_3_O_4_: Iron oxide
GO: Graphene oxide
HNSCC: Head and neck squamous cell carcinoma
HER2: Human epidermal growth factor receptor 2
HSPA2: Heat shock-related 70 kDa protein 2
IC50: Half maximal inhibitory concentration
IL1RAP: Interleukin 1 receptor accessory protein
INSF: Iran National Science Foundation
KLF4: Krüppel-like factor 4
LGR5: Leucine-rich repeat-containing G-protein coupled receptor 5
LNC: Lipid nanocapsule
MgO: Magnesium oxide
Mn_3_O_4_: Manganese oxide
MRI: Magnetic resonance imaging
MWCNT: Multi-walled carbon nanotube
NIR: Near-infrared
OCT4: Octamer-binding transcription factor 4
PCOR: Protocadherin-related receptor
PDT: Photodynamic therapy
PEG: Polyethylene glycol
PEGMA: Poly(ethylene glycol) methacrylate
PLGA: Poly(lactic-co-glycolic acid)
Poly(DEGMA-co-PEGMA): Poly(diethylene glycol methacrylate-co-polyethylene glycol methacrylate)
PTX: Paclitaxel
RNA: Ribonucleic acid
ROS: Reactive oxygen species
SCA-1: Stem cell antigen-1
SOX2: SRY-box transcription factor 2
SOX9: SRY-box transcription factor 9
SPION: Superparamagnetic iron oxide nanoparticle
TME: Tumor microenvironment
TiO_2_: Titanium dioxide
ZnO: Zinc oxide
